# Hemispheric dominance in reading system alters contribution to face processing lateralization across development

**DOI:** 10.1016/j.dcn.2024.101418

**Published:** 2024-07-22

**Authors:** Xinyang Liu, Danni He, Miaomiao Zhu, Yinghui Li, Longnian Lin, Qing Cai

**Affiliations:** a Key Laboratory of Brain Functional Genomics (MOE & STCSM), Affiliated Mental Health Center (ECNU), Institute of Brain and Education Innovation, School of Psychology and Cognitive Science, East China Normal University, Shanghai 200062, China; b Shanghai Changning Mental Health Center, Shanghai 200335, China; c Shanghai Center for Brain Science and Brain-Inspired Technology, East China Normal University, China; d NYU-ECNU Institute of Brain and Cognitive Science, New York University, Shanghai, China; e School of Life Science Department, East China Normal University, Shanghai 200062, China

**Keywords:** Lateralization, Face processing, Reading, Children, Development

## Abstract

Face processing dominates the right hemisphere. This lateralization can be affected by co-lateralization within the same system and influence between different systems, such as neural competition from reading acquisition. Yet, how the relationship pattern changes through development remains unknown. This study examined the lateralization of core face processing and word processing in different age groups. By comparing fMRI data from 36 school-aged children and 40 young adults, we investigated whether there are age and regional effects on lateralization, and how relationships between lateralization within and between systems change across development. Our results showed significant right hemispheric lateralization in the core face system and left hemispheric lateralization in reading-related areas for both age groups when viewing faces and texts passively. While all participants showed stronger lateralization in brain regions of higher functional hierarchy when viewing faces, only adults exhibited this lateralization when viewing texts. In both age cohorts, there was intra-system co-lateralization for face processing, whereas an inter-system relationship was only found in adults. Specifically, functional lateralization of Broca’s area during reading negatively predicted functional asymmetry in the FFA during face perception. This study initially provides neuroimaging evidence for the reading-induced neural competition theory from a maturational perspective in Chinese cohorts.

## Highlights

• Face processing and reading systems lateralize among school-aged children.
• Higher-level functional regions show larger asymmetry in face and text processing.
• Face-related functional areas co-lateralize in both children and adults.
• Asymmetry in face processing is associated with reading only in adults not children.

## 1 Introduction

Human brain development is accompanied by the maturation of functional specialization, presented as selective neural activation in certain brain regions when performing specific perceptual or cognitive tasks (Golarai et al., 2007, Dobs et al., 2022). For many cognitive abilities, one hemisphere plays a dominant role in neural processing compared to the other side, known as hemispheric or functional lateralization (Gotts et al., 2013, Güntürkün et al., 2020). On the one hand, functional specialization and lateralization optimize brain space and computational efficiency, thus allowing for parallel execution of multiple tasks and improvement in cognitive capacities (Güntürkün et al., 2020, Rogers, 2021). On the other hand, different functions may influence each other in cortical distribution, such as the interaction between lateralization in language processing and visuospatial attention (Cai et al., 2010, Cai et al., 2013, Esteves et al., 2020, Gerrits et al., 2020). Furthermore, such inter- or between-system effects on hemispheric lateralization may differ across cognitive functions, brain regions and individual developmental sessions, the mechanisms of which remain to be figured out. In the current study, we aimed to concretely investigate how the lateralization of the core face processing system is influenced by that of the reading system and how the pattern of influence varies across different age cohorts and hierarchical brain regions.

Face cognition, a set of specific abilities to perceive and recognize faces, is crucial in daily social activities (Wilhelm et al., 2010, Liu et al., 2017, Liu et al., 2020). During the processes of multidimensional facial information, a right hemisphere dominance has been extensively evidenced among typical and atypical populations (Rossion and Jacques, 2011, Cohen et al., 2019; Murty et al., 2021) using various techniques (Yovel et al., 2003, Brederoo et al., 2020, Jonas and Rossion, 2021). While this functional asymmetry elucidates the nature of brain functional specialization and associates with face cognitive performances (Meng et al., 2012, Frässle et al., 2016, Dahlén et al., 2022), the reason why face processing dominates the right side of the brain in most individuals is still unclear (for reviews see Behrmann and Plaut, 2020; Rossion and Lochy, 2022).

Several influential theories in the domain of hemispheric lateralization provide potential explanations. First, the *causal complementarity theory* (Bryden et al., 1983, Bryden, 1990) postulates that the lateralization of a certain cognitive function may be caused by the growing asymmetry of its complementary functional system which produces neural inhibition to homologous brain regions. Second, the *statistical complementarity theory* presumes that asymmetrical functional distribution of a cognitive system can be irrelevant of other functions, but comes from a probabilistic bias that a functional system has a likelihood of being lateralized in one hemisphere (Bryden et al., 1983, Bryden, 1990). Third, lower-level brain functional asymmetry can also give rise to the lateralization of higher-level cognitive functions. Specifically, if lower-level input is processed more efficiently in one hemisphere, higher-level processing can benefit from operating more efficiently in the same hemisphere. This concept is known as the *input asymmetry theory* (Sergent, 1982, Sergent, 1983, Andresen and Marsolek, 2005).

As a concrete case of the causal complementarity theor*y*, a long-standing debate exists on whether the acquisition of reading skills primarily determines the right hemisphere dominance of face cognition (for review, see Rossion and Lochy, 2022). When viewing texts, the visual word form area (VWFA), posterior superior temporal gyrus (pSTG) and Broca’s area are respectively responsible for visual text processing (Dehaene and Cohen, 2011), phonological-orthographic integration (Blomert, 2011) and syntactic and semantic processing (Musso et al., 2003). Specifically, the VWFA generates in the ventral occipital-temporal cortex (vOTC) during reading acquisition and reuses its neighboring functionally-similar regions, especially the face fusiform area (FFA; Kanwisher et al., 1997). Since the cortical distribution of VWFA is driven by the language system to be left-lateralized (Cai et al., 2008, Cai et al., 2010), neural competition is deemed to occur between the VWFA and FFA in the left vOTC, prompting the rightward asymmetry of face cognition (Dehaene et al., 2015, Behrmann and Plaut, 2015, Behrmann and Plaut, 2020). Despite the great influence of reading-induced lateralization in face processing, several recent studies reported that learning to read did not damage or inhibit face responses, but generally enhanced neural representation in the visual cortex (Hervais-Adelman et al., 2019; Paridon et al., 2021). Additional brain and behavioral studies found reading acquisition exerted no influence on face cognition, but on visual processing of other stimuli such as tools and limbs (for review, see Rossion and Lochy, 2022; Kubota et al.,2023). Therefore, empirical studies from old and new perspectives are needed to figure out relevant underpinnings of the mixed results.

To achieve the goal, one way is to investigate whether the relationship between the face processing system and reading system changes across individual development. Previous studies have revealed distinct developmental features of the VWFA and FFA, with the word-selective region becoming more shrunken and less activated from children to adults (Siok et al., 2020), while the face-selective region getting larger and more responsive (Natu et al., 2016). Such dynamic properties would probably lead to a variation in the inter-system relationship between reading and face processing. However, apart from some longitudinal studies purely based on preschool and school-aged children (Dehaene-Lambertz et al., 2018, Feng et al., 2022) or adults (Braga et al., 2017), few studies compared the relationship difference between the immature and mature cohorts using the same experimental paradigm.

Apart from inter-hemispheric effects, intra-hemispheric interactions may also play a role in prompting functional lateralization in face processing. In line with the input asymmetry theory, previous research revealed that the asymmetry of lower-level visual perception, such as the right-lateralized holistic and low spatial frequency information processing, may partly drive facial information to be processed in the right hemisphere (Rossion, 2013, Quek et al., 2018, Robertson, 2020). Similarly, although not sufficiently examined in previous research, face-selective regions at different functional hierarchies possibly have intra- or within-system interactions for a general co-lateralization. Specifically, in the core face processing system (Haxby et al., 2000), the occipital face area (OFA) provides input of early visual features to the fusiform face area (FFA) and posterior superior temporal sulcus (pSTS), which respectively analyze invariant and changeable facial information at a higher level. Therefore*,* the lateralization of OFA probably exerts an influence on the lateralized FFA and pSTS.

However, limited studies investigated within-system interactions concerning the lateralization of face-selective cerebral regions, the results of which were inconsistent. A few fMRI studies reported the presence of intra-hemispheric correlations in the core face system (Pinel et al., 2015, Frässle et al., 2016, Canário et al., 2020). Yet, a recent study based on more than one hundred adults found no significant correlation between the lateralization degree of the FFA, OFA and STS (Thome et al., 2022). Additionally, despite the important role of pSTS in the face system (Duchaine and Yovel, 2015) and its right-lateralized functional distribution (De Winter et al., 2015, Sliwinska and Pitcher, 2018), potential reasons to drive its hemispheric asymmetry have been largely neglected in previous research as compared with the overwhelmingly discussed FFA in the ventral visual pathway. Therefore, a complementary exploration is needed.

It is important to note that, although cross-cultural reading systems are highly universal (Martin et al., 2016, Feng et al., 2020), the logographic Chinese script is unique due to its graphic representation and holistic processing of Chinese characters (Guo et al., 2022, Ma et al., 2022). A recent study has demonstrated that Chinese reading exhibits greater neural representation overlap with face processing compared to alphabetic reading (Zhan et al., 2023). Considering the overwhelming proportion of alphabetic scripts in previous neural competition studies and the merely existed psychophysiological studies about the Chinese script system (Li et al., 2013, Fan et al., 2015, Cao et al., 2019), it is important to carry out neuroimaging studies to examine whether specificity also exists in the relationship between lateralization in face processing and Chinese reading.

In this study, we aim to investigate both intra- and inter-system influences on the right lateralization of core face processing system from the developmental and hierarchical perspectives. We focused on three functional regions of interest (ROIs) within the core face system namely the OFA, FFA and pSTS and three crucial regions within the reading system which were the VWFA, pSTG and Broca’s area. Both children and adults were instructed to finish a passive-viewing task with multiple types of stimuli. The weighted bootstrapped lateralization indices (LIs) were computed as the primary indicators for all target ROIs.

## 2 Methods

### 2.1 Participants

Forty adults (30 females, mean age = 22.48 years, range 18–29 years) and thirty-six children (16 females, mean age = 9.86 years, range 9–11 years) were recruited in the current study. All participants were right-handed native Chinese speakers with normal or corrected-to-normal visual acuity. They had no history of neurological or psychiatric disorders. Child participants came from local elementary schools and adult participants were college students. Adult participants and parents of children provided written informed consent approved by the East China Normal University Institutional Review Board (HR142–2018). All the children were asked for their assent.

### 2.2 Stimuli and experimental design

**Fig. 1:**
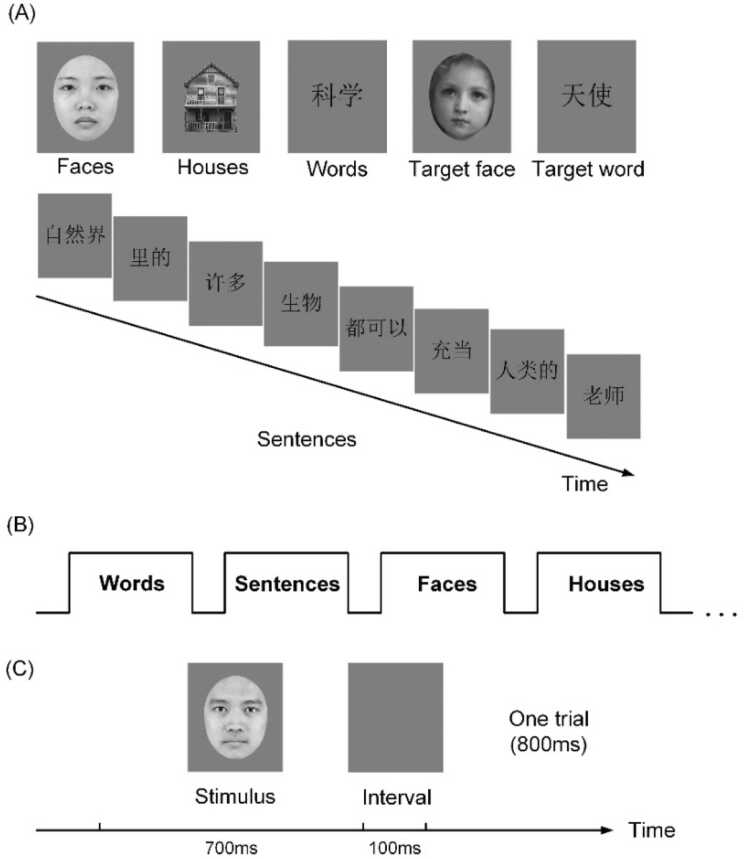
Visual stimuli and experimental design. (A) Examples of stimuli in the passive viewing fMRI task. (B) Block design in the experiment. (C) Trial structure in each block.

Participants completed a passive-viewing functional MRI experiment during which cortical responses to different visual stimuli were recorded. Four categories of stimuli were displayed on the screen during the scan, namely faces, words, sentences and houses (see Fig. 1). Sixty-four pictures of Chinese young adults’ faces were selected from the Tsinghua facial expression database (Tsinghua-FED, Yang et al., 2020) with sex balance controlled for. All external facial features including hair, neck, and chin shape were masked by a gray oval. Twenty-five houses were chosen from the DalHouses database (Filliter et al., 2016). Four articles from popular science books for children were used as materials for visual reading. They were matched for word frequency, sentence length, readability, syntactic features, and semantic features. Altogether, we selected 18 short sentences, each of which was segmented into eight components consisting of one or two Chinese words (1–3 characters). These components were sequentially presented within a block for coherent sentence reading. A total of 144 Chinese words from the articles were selected for word reading, including examples such as 计划(plan), 人类(human) and速度(speed). The ratio of words and sentences from each article were matched.

All visual stimuli were converted to grayscale with identical brightness and contrast. The experimental screen was 89×50 cm with a resolution of 1920 ×1080 pixels. Participants watched the stimuli through a mirror from a distance of 164 cm. The whole experiment contained three runs. In each run, a fixation cross was first shown on the screen for 10 s. Then, eight passive-viewing blocks were exhibited in a pseudo-random sequence, with each category of visual stimuli displayed twice. A single block for either faces, words or houses consisted of 25 trials. A sentence block comprised 24 trials for two Chinese sentences, which were separated by two fixation intervals. One visual stimulus was presented for 700 ms per trial followed by a 100 ms blank screen (See Fig. 1C). An inter-sentence fixation interval lasted for 400 ms. Each experimental block had a duration of 20 s, followed by a 10-s blank screen. Ten more brain volumes were collected at the end of each run to capture complete information. Participants were instructed to silently view the stimuli without providing any behavioral responses. To guarantee a constant focus, a target trial showing an angel’s face or a Chinese word meaning angel was displayed in two of the three runs, as shown in Fig. 1A. After each session, participants should report the number of angel trials. In total, the duration of an entire run amounted to 260 s.

### 2.3 Imaging acquisition

The experiment was conducted based on a 3 T Siemens Prisma scanner (Siemens, Erlangen, Germany) with a 64-channel head coil. E-prime3 was used for stimuli presentation. All participants were protected by wearing noise-cancelling earphones. Children underwent training in a mock scanner before the real image acquisition. During the experiment, T1 images were first obtained using a MPRAGE sequence (voxel size = 1×1×1 mm, TR = 2300 ms, TE = 2.25 ms, FA = 8 deg). Then functional images were collected using an inter-leaved multiband echo planar imaging (EPI) sequence (voxel size = 2×2×2 mm, TR = 1000 ms, TE = 32 ms, FA = 55 deg). Each run acquired 260 volumes. To correct possible distortions of EPI images, a pair of field maps were collected using a gradient recalled echo (GRE) sequence (voxel size = 3×3×3 mm, TR = 413 ms, TE1 = 4.92 ms, TE2 = 7.38 mm, FA = 60 deg). Image quality was checked after each scan.

### 2.4 Preprocessing of brain images

MRI data preprocessing was performed using SPM12 (http://www.fil.ion.ucl.ac.uk/spm/). Initially, voxel displacement maps (VDMs) were calculated based on the field mapping images and subsequently applied to the EPI images for unwarping. Then, the functional images were realigned to the first volume in each run, corrected for susceptibility-induced distortion, and adjusted for slice timing difference. After manually setting the anterior commissure of T1 images as the origin, we co-registered the individual functional image to its corresponding anatomical image and normalized it to the MNI space. Finally, a 4 mm full-width at half-maximum (FWHM) Gaussian kernel was applied for spatial smoothing.

To detect bad volumes and assist in better model estimation, the ARtifact detection Tools (ART; https://www.nitrc.org/projects/artifact_detect/) was applied before the first-level analysis. Preprocessed functional images were identified and recorded as outliers either if their global brain activation deviated by more than 9 SD from the mean value within a single run, or if their linear scan-to-scan head movement exceeded 5 mm.

### 2.5 First-level analysis

For each participant, we performed first-level analysis with preprocessed functional images across runs using a General Linear Model (GLM; Friston et al., 2005). The convolution of canonical hemodynamic response function (HRF) in SPM and the four experimental conditions were modeled as regressors. Outliers and head motion information from the ART processing were modeled as regressors of no interest. To obtain category-specific neural activities, we generated global maps using *Faces* versus *Baseline* for face-selective activity, *Words* versus *Baseline* for word-selective activity, and *Sentences* versus *Baseline* for high-level sentence processing activity. Here the baseline indicated the mean of neural signal fluctuations over all scans. Notably, we hadn’t used the *house* condition as a planned contrast base due to its emerged right hemispheric dominance in target ROIs introduced below (see Figure S1).

### 2.6 Definition of ROIs

Target ROIs were defined at both hemispheres by taking the intersection of an anatomical mask and a functional mask. The anatomical masks were extracted from the AAL3 atlas (Rolls et al., 2020). The functional masks were 20 mm radium spheres manually drawn based on peaks from Neurosynth meta-analyses (https://neurosynth.org/; Yarkoni et al., 2011). We searched the keyword *face* to identify the FFA, OFA and pSTS in the face processing system, and *word* for the VWFA, *sentence* for the pSTG and Broca’s area in the reading system. Spatial coordinates of Neurosynth-defined functional peaks are listed in Table 1. Considering the close spatial location and large overlap of the FFA and VWFA in the ventral occipitotemporal cortex (vOTC), which was not only found in both Neurosynth and our pilot analyses but also displayed in previous studies (Behrmann and Plaut, 2020, Canário et al., 2020, Feng et al., 2022), these two regions were merged into one ROI named vOTC. Similarly, the pSTS for face processing and pSTG for reading were encompassed within a consolidated ROI referred to as pSTS/G (Beauchamp, 2015). Besides, since the Broca’s area is typically defined as a combination of the pars opercularis and pars triangularis (Dronkers et al., 2007), we correspondingly created two Neurosynth-defined ROIs and merged them within the anatomical constrain. Ultimately, we created four pairs of pre-defined ROIs to investigate face and reading related activities. All of them were bilaterally symmetrical. For expression convenience, we still used the name FFA for neural response to faces in the vOTC, VWFA for neural response to word reading in the vOTC, pSTS for neural response to faces in the pSTS/G, and pSTG for neural response to sentence reading in the pSTS/G in subsequent analyses.

**Table 1:** Detailed information about pre-defined ROIs.

Target ROI	Anatomical mask	Functional mask	Hemisphere	MNI coordinates of the Neurosynth-defined peak voxel		
				x	y	z
vOTC	Fusiform_gyrus, Temporal_Inf	FFA	Left	−40	−52	−22
			Right	40	−50	−20
		VWFA	Left	−42	−44	−14
			Right	48	−60	−10
OFA	Occipital_Inf	OFA	Left	−38	−86	−14
			Right	44	−76	−14
pSTS/G	Temporal_Mid, Temporal_Sup	pSTS	Left	−54	−56	8
			Right	54	−40	6
		pSTG	Left	−52	−42	4
			Right	48	−32	4
Broca	Frontal_Inf_Tri, Frontal_Inf_Oper	Broca_Tri	Left	−54	22	18
			Right	50	22	22
		Broca_Oper	Left	−52	18	14
			Right	54	20	28

*Note:* Temporal_Inf - Inferior temporal gyrus; Occipital_Inf - Inferior occipital gyrus; Temporal_Mid - Middle temporal gyrus; Temporal_Sup - Superior temporal gyrus; Frontal_Inf_Tri - Inferior frontal gyrus, triangular part; Frontal_Inf_Oper - Inferior frontal gyrus, opercular part; Broca_Tri – Part of the Broca’s area in the Frontal_Inf_Tri; Broca_Oper - Part of the Broca’s area in the Frontal_Inf_Oper.

### 2.7 Calculation of hemispheric lateralization

Conventionally, functional lateralization was estimated by the lateralization index (LI) using the formula: LI = (L-R)/(L+R), where L and R separately indicated brain activation in the left and right hemispheres (Binder et al., 1996, Seghier, 2008). Ranging between −1 and 1, positive and negative LIs respectively signify left and right hemisphere advantage, with larger absolute values reflecting stronger lateralization. Despite a widespread use, this simple algorithm has been criticized for a low reliability attributed to its sensitivity to threshold selection and susceptibility to statistical outliers (Wilke and Schmithorst, 2006).

To enhance methodological robustness, we used the LI toolbox (Wilke and Lidzba, 2007) based on SPM12. The bootstrap approach (Wilke and Schmithorst, 2006) was employed for the calculation of weighted LIs. Functional asymmetry was indicated by the number of voxels exceeding a specified threshold within each ROI. Category-selective brain activities in corresponding target ROIs were computed to derive LIs. Specifically, the global t-map of *Faces* versus *Baseline* was analyzed in the vOTC, OFA and pSTS/G to obtain weighted LIs of the face-related FFA, OFA and pSTS. In terms of reading, the global t*-*map of *Words* versus *Baseline* was analyzed in the vOTC to compute the weighted LI of VWFA, and the t-map of *Sentences* versus *Baseline* was used to compute weighted LIs of the pSTG and Broca’s area. The brain region within 5 mm off the midline was employed as an exclusive mask to remove potential flow artifacts.

As a comparable indicator, the percent signal change (PSC) in response to faces and texts was also computed. While LI is an integrate metric to estimate hemispheric difference, the magnitude of neural activation in PSC computation can provide information from another perspective that whether single hemispheres display a distinctive pattern in intra- and inter-system relationships concerning functional lateralization.

**Fig. 2:**
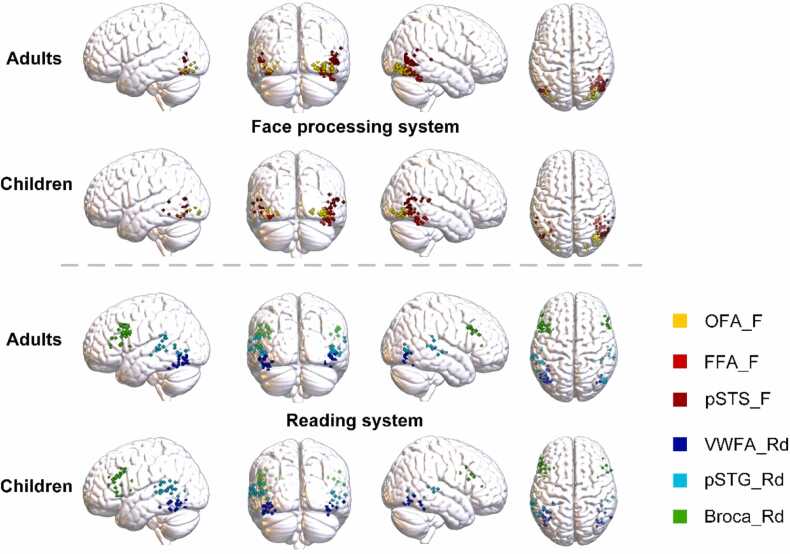
Maximumly activated points within functionally dominant target ROIs for all individuals. Yellow, red and dark red points correspondingly signify brain activation peaks in the dominant hemisphere of the OFA, FFA and pSTS in the face processing system. Blue, light blue and green points separately denote neural responses in the dominant hemisphere of the VWFA, pSTG and Broca’s area in the reading system.

For each child and adult participant, the largest neural responses to faces, words and sentences in bilateral target ROIs were visualized by showing peak points of their t-maps in the dominant hemisphere (see Fig. 2). All participants exhibited significant neural activation in the target ROIs (*p*<0.05, FWE corrected). The computation and visualization were respectively based on the Marsbar toolbox for SPM (http://marsbar.sourceforge.net) and MRIcroGL (https://www.mccauslandcenter.sc.edu/mricrogl).

### 2.8 Statistical analyses

First, we assessed the functional lateralization of face processing and reading in each age group. One-sample Wilcoxon signed rank test was applied to the estimation of weighted mean LIs given non-normal distribution of the data. Comparing to zero, significant positive or negative LI values separately indicated left or right hemispheric dominance.

Second, the effects of age and region on lateralization were separately estimated in face processing and reading systems using 2×3 mixed-designed ANOVA. Age was involved (children vs. adults) as a between-subject factor, and the category-related ROI was computed as a within-subject factor (FFA vs. OFA vs. pSTS for face processing, and VWFA vs. pSTG vs. Broca for reading). Given the non-normally distributed LIs as the dependent variable, a robust rank-based ANOVA-type statistic (ATS) was applied for the factorial setting using the nparLD package in R (Noguchi et al., 2012). This nonparametric method has been proven to work well with data in violation of the prerequisite of ANOVA (Brunner et al., 2017).

Finally, we investigated inter- and intra-system influences on lateralization of the FFA and pSTS as functionally higher-level ROIs in the core face processing system. For an initial overview, we computed Spearman correlation matrices of LIs in all category-selective ROIs to examine relationships within and across the two systems. Then, we conducted path analysis based on the LIs of all ROIs using the lavaan package (Rosseel, 2012) in R (R Core Team, 2016). Weighted mean LIs of the FFA and pSTS during visual face perception were respectively set as endogenous variables in two models, while the LIs from the rest two face-related ROIs and three reading-related ROIs were modelled as exogenous variables. Spearman correlation matrices of LIs entered the models as input. Notably, considering the skewed LI distribution, path analysis supplanted multiple regression to examine multivariate relationships, which has been proved as a powerful extension (Streiner, 2005). Besides, power analysis was carried out by conducting the Monte Carlo simulation approach (Muthén and Muthén, 2002) using the pwrSEM app in R (Wang and Rhemtulla, 2021). Given a sample size of 80, our hypothesized model with one endogenous variable and five exogenous variables estimated by maximum likelihood would have a power of 0.78 to detect a target effect of 0.3 at the alpha level of 0.05, and a power of 0.99 to detect a target effect of 0.5. When the sample size was 40, the model would have a power of 0.83 to detect an effect size of 0.4. Both correlation computation and path analyses were conducted respectively among adults, children and all participants for age comparison. All statistical analyses were performed using the R software (R Core Team, 2016).

## 3 Results

### 3.1 Functional asymmetries in face- and reading-related ROIs

**Fig. 3:**
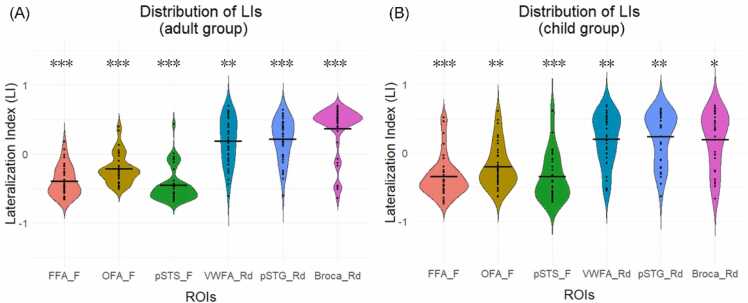
Distribution of weighted mean LI values in the adult and child groups. (A) LI distribution of all target ROIs in the adult group. (B) LI distribution of all target ROIs in the child group. FFA_F, OFA_F, pSTS_F represent three core regions in the face processing system. VWFA_Rd, pSTG_Rd and Broca_Rd indicate three crucial ROIs in the reading system. *p< 0.05, ** *p* < 0.01, *** *p* < 0.001, FDR corrected.

All face-related ROIs exhibited significant right hemispheric dominance for both adults (OFA: *Z*= −4.36, FDR *q* < 0.001, *r* = 0.69; FFA: *Z* = −5.37, FDR *q* < 0.001, *r* =0.85; pSTS: *Z* = −5.36, FDR *q* < 0.001, *r* = 0.85) and children (OFA: *Z* = - 3.17, FDR *q* < 0.01, *r* = 0.53; FFA: children: *Z* = −4.32, FDR *q* < 0.001, *r* = 0.72; pSTS: *Z* = −4.09, FDR *q* < 0.001, *r* = 0.68). Similarly, all reading-related ROIs reached significant functional lateralization to the left hemisphere, including the adult group (VWFA: *Z* = −3.08, FDR *q* <0.01, *r* = 0.49; pSTG: *Z* = −3.72, FDR *q* <0.001, *r* = 0.59; Broca: *Z* = −4.3, FDR *q* < 0.001, *r* = 0.68) and the child group (VWFA: *Z* = −2.94, FDR *q* < 0.01, *r* = 0.49; pSTG: *Z* = −3.33, FDR *q* <0.01, *r* = 0.56; Broca: *Z* = −2.43, FDR *q* < 0.05, *r* = 0.41). Visualized LI distributions are shown in Fig. 3.

### 3.2 Age and regional effect on the hemispheric dominance

**Fig. 4:**
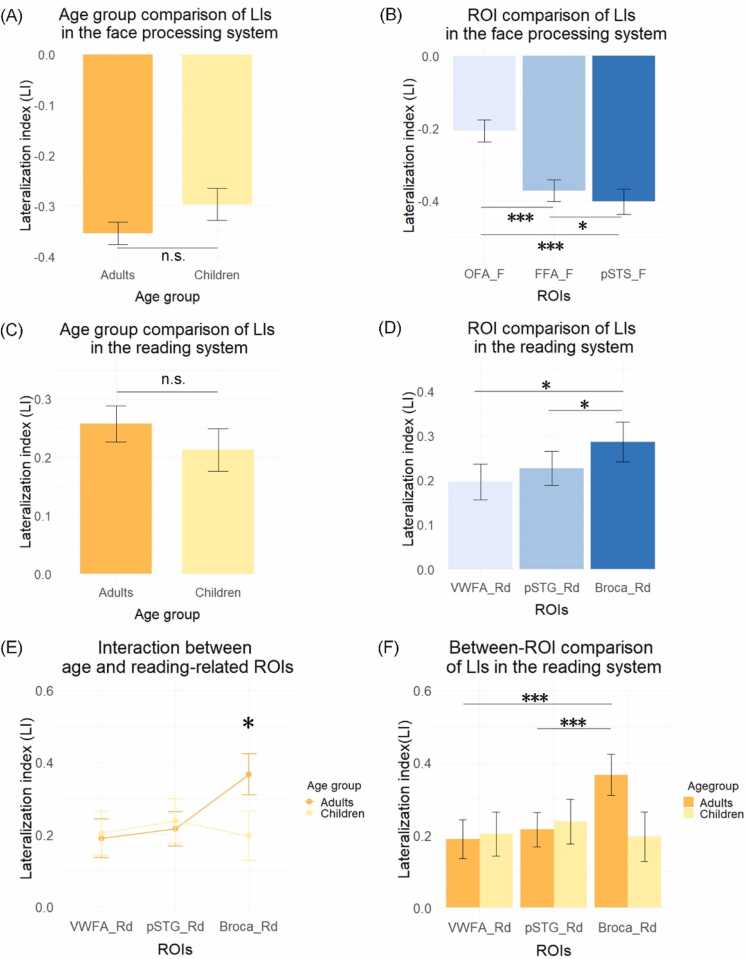
Main effects and interactions of age and ROIs on functional lateralization in the face processing and reading systems. (A) Age difference of the LI in the core face processing system. (B) ROI difference of the LI in the core face processing system. (C) Age difference of the LI in the reading system. (D) ROI difference of the LI in the reading system. (E) Interaction between age and ROIs in the reading system. (F) Contrasts between functional lateralization of different ROIs in the reading system separately among adults and children. OFA_F, FFA_F, pSTS_F represent three core regions in the face processing system. VWFA_Rd, pSTG_Rd and Broca_Rd indicate three crucial ROIs in the reading system. *p< 0.05, ** *p* < 0.01, FDR corrected.

In the core face processing system, ROIs (*F* = 29.0, *df* = 1.79, *p* < 0.001) but not age (*F* = 0.37, *df* = 1, *p* = 0.54) had a significant effect on the weighted mean LIs (see Fig. 4A and B). There was no significant interaction between these two factors (*F* = 1.93, *df* = 1.79, *p* = 0.15). Post hoc analyses for the main effect of ROIs using the same statistical method further revealed the mean LIs were higher in the FFA than OFA (*F* = 37.0, *df* = 1, FDR *q* < 0.01), and higher in the pSTS than both the OFA (*F* = 37.9, *df* = 1, FDR *q*< 0.01) and the FFA (*F* = 6.06, *df* = 1, FDR *q* = 0.014), as marked in Fig. 4B. Therefore, face-selective ROIs at a higher functional level are generally more lateralized to the right hemisphere than the lower level area.

In the visual reading system, there was a significant main effect of ROIs (*F* = 4.92, *df* = 1.98, *p* < 0.01) but not age (*F* = 0.18, *df* = 1, *p* = 0.67) on the degree of lateralization (see Fig. 4C and D). The interaction effect of ROI and age was significant (*F* = 5.4, *df* = 4.98, *p* < 0.01), indicating that functional asymmetry of reading-related ROIs differed between children and adults (Fig. 4E). Post hoc analysis using the same statistical method further revealed that only adults had significantly larger degree of lateralization in the Broca’s area than the pSTG (*F* = 16.6, *df* =1, FDR *q* < 0.001) and VWFA (*F* = 11.73, *df* =1, FDR *q* < 0.001), displayed in Fig. 4F. For children, there was no between-ROI difference. Besides, adults had stronger lateralization in the Broca’s area than children during reading (*W* = 918, *p* = 0.039, FDR *q* = 0.12, Wilcoxon rank-sum test; Fig. 4E).

Together, these results indicated that in passive viewing of faces, the functional lateralization of the OFA, FFA and pSTS among school-aged children already reached a similar level to adults. Besides, lateralization of the lower-level OFA is less pronounced than the higher-level FFA and pSTS. For the passive reading of words and sentences, similar hierarchical feature of functional lateralization in the VWFA, pSTG and Broca’s area was displayed in adults but not school-aged children.

### 3.3 Relationships between lateralization indices of the face and reading-related ROIs

**Fig. 5:**
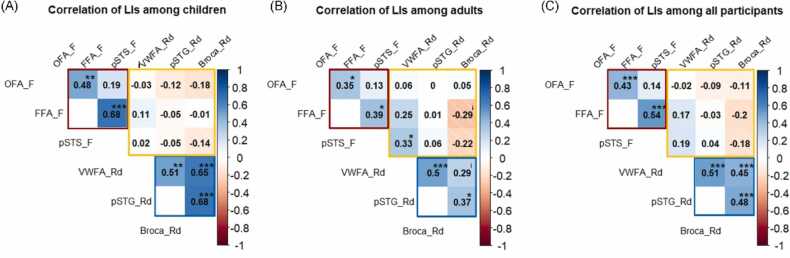
Correlation matrices for the LIs of different ROIs in the core face and reading system. (A) The LI correlation matrix for all the ROIs in the adult group. (B) The LI correlation matrix for all the ROIs in the child group. (C) The LI correlation matrix for all the ROIs among all participants. FFA_F, OFA_F, pSTS_F represent the LIs of three core regions in the face processing system. VWFA_Rd, Broca_Rd and pSTG_Rd indicate the LIs of three crucial ROIs in the reading system. The red and blue boxes separately cover the LI correlations within the face processing and reading systems. The yellow box involves inter-system LI relationships between face-selective and reading-related ROIs. *p< 0.05, ** *p* < 0.01, *** *p* < 0.001, uncorrected for illustration purpose.

To estimate the relationship between the LIs of different ROIs in the face and reading systems, we performed Spearman correlation analyses separately for children, adults and all the participants. The results are visualized as correlation matrices in Fig. 5. Among children, functional lateralization in the face processing system and reading system exhibited a clear independency. Respectively, the LIs of the FFA had a significant correlation with the OFA ($r_{s}$ = 0.48, *p* < 0.01, FDR *q* < 0.01) and the pSTS ($r_{s}$ = 0.68, *p* < 0.001, FDR *q* < 0.001) within the face system. And there existed significant relationships between the LIs of the Broca’s area and pSTG ($r_{s}$ = 0.68, *p* < 0.001, FDR *q* < 0.001), the Broca’s area and VWFA ($r_{s}$ = 0.65, *p* < 0.001, FDR *q* < 0.001), as well as the pSTG and VWFA ($r_{s}$ = 0.51, *p* < 0.01, FDR *q* < 0.01) in the reading system. No across-system relationships were found between face-related and reading-related ROIs.

Comparably, intra- and inter-system relationships of LIs both existed in the adult group. Similar with children, functional lateralization of the FFA was positively related with that of the OFA ($r_{s}$ = 0.35, *p* = 0.025, FDR *q* = 0.09) and pSTS ($r_{s}$ = 0.39, *p* = 0.013, FDR *q* = 0.09) during face processing, and the pSTG significantly co-lateralized with the VWFA ($r_{s}$ = 0.50, *p* < 0.01, FDR *q* = 0.015) and Broca’s area ($r_{s}$ = 0.37, *p* = 0.02, FDR *q* = 0.09) in passive reading. Furthermore, there was a significant inter-system relationship between the lateralization of pSTS and VWFA ($r_{s}$ = 0.33, *p* = 0.036, FDR *q* = 0.108), and a marginally significant relationship between the FFA and Broca’s area ($r_{s}$ = −0.29, *p* = 0.065, FDR *q* = 0.15). Therefore, apart from within-system bindings, the core face processing and reading systems had interactions in terms of hemispheric lateralization among adults.

When involving all participants, the pattern of within-system LI correlations showed similarity to the child group. For the face processing system, lateralization of the FFA was significantly correlated with both the OFA ($r_{s}$ = 0.43, *p* < 0.001, FDR *q* < 0.001) and pSTS ($r_{s}$ = 0.54, *p* < 0.001, FDR *q* < 0.001). While the LIs of the three ROIs in the reading system all had crucial relationships, including the VWFA and Broca’s area ($r_{s}$ = 0.48, *p* < 0.001, FDR *q* < 0.001), the VWFA and pSTG ($r_{s}$ = 0.51, *p* < 0.001, FDR *q* < 0.001), as well as the Broca’s area and pSTG ($r_{s}$ = 0.45, *p* < 0.001, FDR *q* < 0.001). There was no significant relationship between the two cognitive systems.

### 3.4 Path models estimating influential factors to the lateralization of face processing

To examine potential influential factors contributing to the right hemisphere dominance of the core face processing system, we performed path analysis based on a series of hypothesized models. Since all the models were saturated with perfect model fit (CFI = 1.00, RMSEA=0.00, SRMR =0.00), we reported path coefficients and their significance rather than fit indices.

**Fig. 6:**
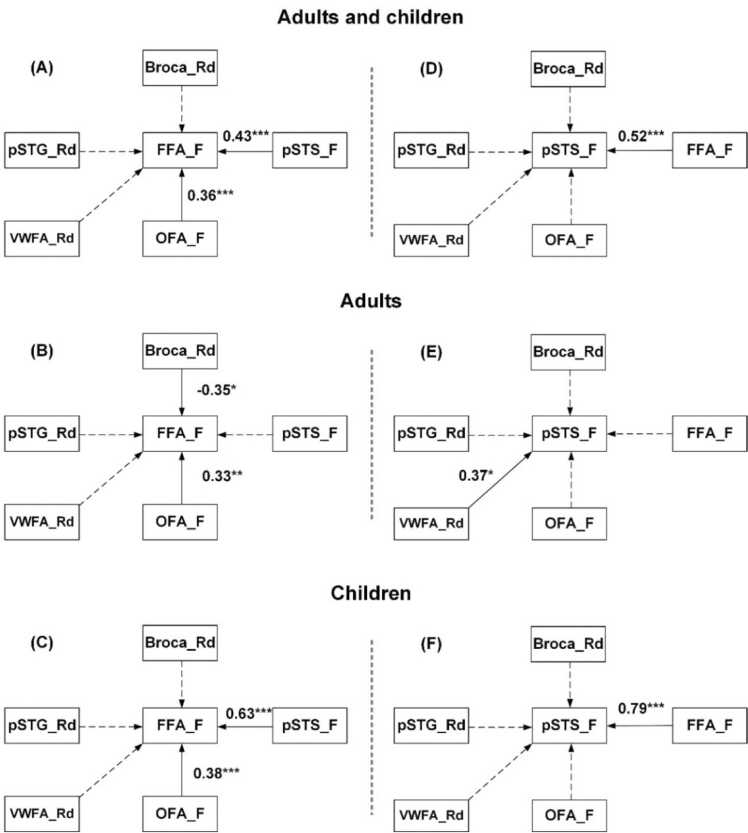
Schematic representations of path models explaining individual differences in the functional lateralization of the FFA and pSTS during face processing. (A) Predictive model for the lateralization of FFA among all participants. (B) Predictive model for the lateralization of FFA among adults. (C) Predictive model for the lateralization of FFA among children. (D) Predictive model for the lateralization of pSTS among all participants. (E) Predictive model for the lateralization of pSTS among adults. (F) Predictive model for the lateralization of pSTS among children. FFA_F, OFA_F, pSTS_F represent the LIs of three core regions in the face processing system. VWFA_Rd, Broca_Rd and pSTG_Rd indicate the LIs of three crucial ROIs in the reading system. *p< 0.05, ** *p* < 0.01, *** *p* < 0.001.

First, we estimated the impact of within- and across-system ROIs on the functional lateralization of the FFA during face perception. When involving all participants, both the OFA (*r* = 0.36, *p* <0.001; *p* = 0.017, permutation test) and pSTS (*r*= 0.43, *p* < 0.001; *p* = 0.01, permutation test) from the core face system had significant influences on the hemispheric dominance of the FFA with medium effect size of around 0.4 (see Fig. 6A). The within-system influences were positive, indicating more right-lateralized OFA and pSTS correlated with a stronger right hemispheric dominance of FFA. Comparably, the inter-system relationship was not found. However, this general pattern differed between the child and adult groups. On the one hand, the positive relationship between the lateralization of FFA and OFA within the core face system both existed among adults (*r* = 0.33, *p* < 0.01; *p* = 0.017, permutation test) and children (*r* = 0.38, *p* < 0.001; *p* = 0.004, permutation test), while a strong relationship between the FFA and pSTS was only found among children (*r* = 0.63, *p* < 0.001; *p* = 0.001, permutation test) when influences from other variables were controlled for. On the other hand, the left hemisphere dominance of the Broca’s area (*r* = −0.35, *p* < 0.01; *p* = 0.023, permutation test) exerted a medium cross-system effect on the right hemisphere dominance of FFA for adults but not children. The modeling results are visualized in Fig. 6B and C.

Second, we further assessed whether the right hemisphere advantage of the pSTS during face processing can be accounted for by potential target regions in the face and reading systems. For all participants, we found only the LIs of FFA (*r* = 0.52, *p* < 0.001; *p* = 0.008, permutation test) play a significant role in predicting the lateralization of the pSTS (see Fig. 6D). Similar to prediction of lateralization in the FFA, the predictive pattern for the pSTS was different between adults and children. To be specific, the large effect from FFA on the LI of pSTS only emerged in the child group (*r* = 0.79, *p* < 0.001; *p* < 0.001, permutation test), as shown in Fig. 6F. Comparably, there existed a strong relationship between the LIs of VWFA during reading and the pSTS in face perception (*r* = 0.37, *p* = 0.028; *p* = 0.046, permutation test) among adults, as shown in Fig. 6E.

In terms of the predictive ability of all hypothesized models, the lateralization of OFA and pSTS in the core face system and the VWFA, pSTG and Broca’s area could together explain 44.5 % of variance in the lateralization of FFA in face processing for all participants, 35.8 % of variance for adults, and 62.3 % for children. While the similar predictive model including intra- and inter-system factors could account for 32.9 % of variance in the lateralization of pSTS in face perception for both adults and children, 26.4 % for adults and 52.9 % for children.

To summarize the above results, individual differences in the right hemispheric lateralization of the FFA and pSTS during face processing can not only be explained by the co-lateralized ROIs within the core face system but also by the lateralization degree of ROIs in the reading system. However, the relationship pattern varied across cohorts of children and adults. Comparably, the reading system exerted a large effect only among adults. For school-aged children, the two functional systems were clearly differentiated with each other, with the lateralization of face-related ROIs merely displayed within-system relationships.

### 3.5 Relationships between percent signal changes of bilateral face and reading-related ROIs

**Fig. 7:**
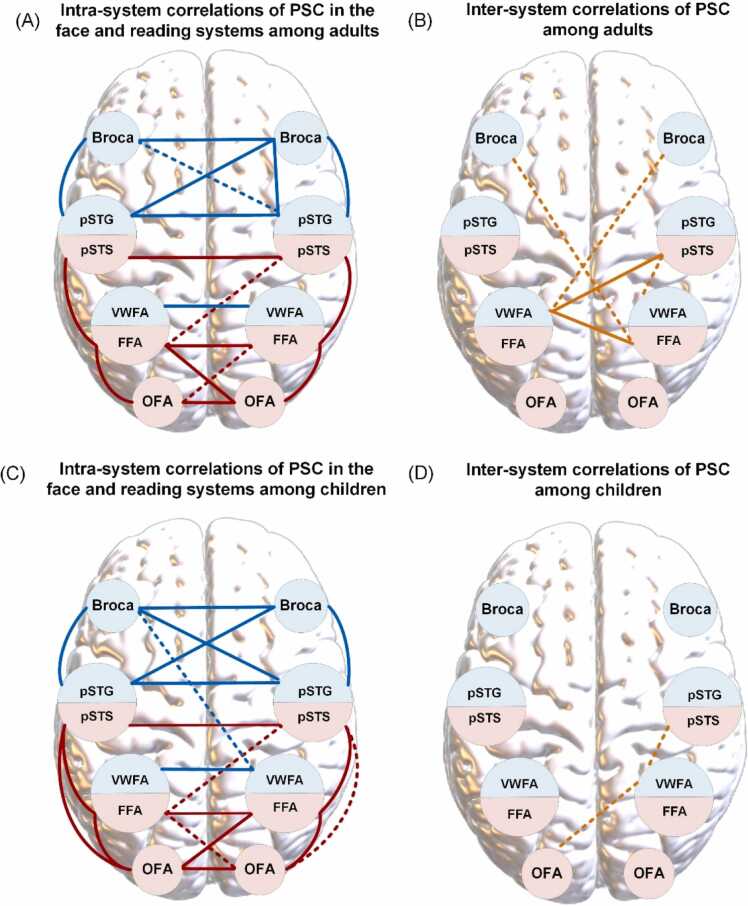
Correlations for PSC of bilateral ROIs in the core face and reading systems. (A) The schematic diagram for intra-system correlations of PSC in the adult group. (B) The schematic diagram for inter-system correlations of PSC in the adult group. (C) The schematic diagram for intra-system correlations of PSC in the adult group. (D) The schematic diagram for inter-system correlations of PSC in the child group. Red lines represent between-ROI correlations of PSC within the core face processing system. Blue lines indicate between-ROI correlations of PSC within the reading system. Orange lines depict inter-system correlations of PSC in the face- and reading-related ROIs. Solid lines denote significant relationships after FDR correction (FDR *q* < 0.05). Dashed lines signify correlations which lost significance after FDR correction (*p* < 0.05 uncorrected, FDR *q* > 0.05).

In addition to analyzing integrated lateralization as above, we also assessed inter- and intra-system correlations using neural activity in single hemispheres. Spearman correlations of the PSC in the left and right target ROIs during face perception and passive reading were calculated. Schematic diagrams are shown in Fig. 7, and the corresponding quantitative results are presented in Tables S1 and S2. As expected, the PSC of system-specific ROIs in bilateral hemispheres exhibited strong correlations in both age cohorts. By contrast, the child group exhibited slightly more intra-system connections than adults, such as the co-lateralized OFA and pSTS during face processing (left hemisphere: *r* = 0.47, *p* < 0.01, FDR *q* = 0.017; right hemisphere: *r* = 0.36, *p* = 0.031, FDR *q* = 0.11). Neural activity in response to faces and written text displayed more inter-system interactions in adults as compared to children. In the adult group, the magnitude of response to written words in the left VWFA exhibited a positive relationship with neural activation to faces in the right FFA (*r* = 0.50, *p* < 0.01, FDR *q* < 0.01) and pSTS (*r* = 0.41, *p* < 0.01, FDR *q* = 0.034). However, only weak relationships existed between PSC of the right hemispheric VWFA during reading and the neural activity of the left OFA (*r* = 0.33, *p* = 0.049, FDR *q* = 0.15) as well as the right pSTS (*r* = −0.36, *p* = 0.033, FDR *q* = 0.11) during face perception, which did not survive after multiple correction.

## 4 Discussion

The present study investigated potential factors that may influence the hemispheric lateralization of face processing system from perspectives of age, functional hierarchy and systematic effects. For both school-aged children and adults, significant functional lateralization in all the target ROIs was observed in responses to faces and Chinese texts. Besides, the ROIs at a higher functional level displayed stronger lateralization, including the FFA and pSTS in the core face system and the Broca’s area in the reading system. But the hierarchical difference in lateralization during passive reading was evident among adults but not children. Furthermore, while strong within-system co-lateralization existed in both age groups, robust across-system interactions between the face processing and reading systems were only found among adults but not children in both the measurements of functional lateralization and neural activation.

### 4.1 Impact of age on lateralization of face and reading systems

Our first main finding was that both school-aged children aged 9–11 years and young adults reached significant functional lateralization in three core face processing ROIs and three crucial reading-related ROIs during passive viewing of faces and visual texts. While there was generally no age effect in both systems, the Broca’s area displayed significantly larger left-hemispheric dominance among adults than children.

In general, the lateralization results converge with the widely known right hemispheric dominance in face processing and left hemispheric advantage in text reading. In terms of face perception, no age effect was found. This is consistent with a series of developmental studies, which reported existed functional asymmetry in core face processing regions among children from 4 to 10 years old (Cantlon et al., 2011, Monzalvo et al., 2012, Dehaene-Lambertz et al., 2018, Feng et al., 2022). Besides, the results can also be explained by a classic viewpoint that simple processing of faces such as visual detection and configural processing get matured earlier in life as compared with difficult tasks such as face memory (McKone et al., 2012, Weigelt et al., 2014). In this way, right hemispheric dominance in passive viewing of faces may already reach the adult level in the middle childhood years.

Notably, a recent cross-sectional fMRI study found hemispheric lateralization of the core face ROIs only among adults but not children (Hildesheim et al., 2020). This is probably due to the strategy to analyze face-selective responses, where house stimuli were set as the contrast baseline. According to the current study using a similar experimental design, significant right hemispheric dominance was found in house-related neural activity. Therefore, a house baseline may lead to biased results to investigate functional lateralization, such as weakened right hemisphere advantage and enhanced left hemisphere advantage (see Figure S2).

As for reading related lateralization, a rapid formation of the VWFA as well as increasing neural response in the left vOTC was found for children during their first year of reading acquisition (Dehaene-Lambertz et al., 2018, Feng et al., 2022). Besides, left-lateralized neural activities in the inferior frontal gyrus (IFG) and superior temporal gyrus (STG) during language comprehension have also emerged in children before 7 years old (Berl et al., 2014, Skeide and Friederici, 2016, Enge et al., 2020). These results can explain our findings that both school-aged children and adults displayed significant left hemispheric dominance in the VWFA, pSTG and Broca’s area. Furthermore, developmental shifts in language lateralization was found to evidently occur in the anterior regions such as the IFG, while changes in posterior language regions was relatively stable (Holland et al., 2007, Berl et al., 2014, Olulade et al., 2020). This is probably due to the enhanced ability in processing syntactic information through maturation (Enge et al., 2020). Therefore, our finding can be supported that the age effect only exhibited in the Broca’s area but not the VWFA and pSTG.

### 4.2 Impact of hierarchical brain regions on lateralization of face and reading systems

Another result in the current study was that the degree of right hemisphere dominance in all the face and reading ROIs changed with the functional hierarchy. For face processing, both the pSTS and FFA in the face processing system displayed stronger lateralization than the OFA, with the pSTS lateralized most. This result is fully consistent with an early fMRI study in which an overall right hemispheric dominance for multiple regions in the face systems was identified, with the pSTS exhibited the strongest lateralization in the core system, followed by the FFA and OFA (Rossion et al., 2012). The present work extended previous finding from adults to school-aged children and therefore verified the phenomenon from a developmental perspective. One potential reason for this finding is that lateralization may increase from the posterior to anterior regions due to an enhanced specialization. However, this hypothesis remains to be tested according to mixed results in limited previous studies (Rossion et al., 2012, Thome et al., 2022).

Similarly, the left hemispheric dominance in the reading system also displayed a gradient feature with the Broca’s area owning the largest degree of lateralization, then the pSTG and VWFA. Besides, this difference only existed among adults but not children. A longitudinal language study showed that lateralization of anterior language areas during story processing and word generation got stronger from childhood to adulthood, while the posterior regions did not have an age-related change in multiple language tasks (Holland et al., 2007). Besides, lateralization of the temporal region in language processing was already developed by age seven, while the functional asymmetry of the frontal area was not strong even from 10 to 12 years and developed until the adult period (Berl et al., 2014; Olulade et al., 2020). These results can together explain our finding that a hierarchical difference in regional lateralization for reading did not exist among children but emerged in adults when the language system gets matured.

Comparatively, distinct asymmetrical patterns in face and text processing emerged during development. This difference is likely influenced by varying cultural factors. Face cognition begins to develop shortly after birth, whereas reading capacity typically develops after children enter elementary school. Therefore, the relatively shorter developmental period for reading may contribute to its differing lateralization compared to face processing.

### 4.3 Intra-system relationships for functional asymmetry in face processing

For within-system interactions in functional lateralization, FFA showed medium to large positive relationships with OFA and pSTS in both measurements of LI and unilateral PSC. Comparably, there was barely no relationship between the OFA and pSTS. These results are generally consistent with neural models of face processing. First, the OFA and FFA belongs to the ventral pathway for processing invariant facial features, and the pSTS locates in the dorsal pathway for processing dynamic facial information (Haxby et al., 2000). This pathway separation was not revealed in functional blocks (Duchaine and Yovel, 2015; Bernstein and Yovel, 2015) but also found in structural connections (Gschwind et al., 2012; Liu et al., 2020, Liu et al., 2022; Sanchez et al., 2021). Additionally, although the OFA is deemed as an input of pSTS in the Haxby model, a modified framework proposed by O'Toole et al. (2002) suggested that the pSTS mainly receives input from the middle temporal (MT) visual area for face processing. Therefore, the distinction in neural routes and input source may together explain the barely existed relationship in lateralization between the OFA and pSTS.

Second, although the FFA and pSTS locate in different functional routes, they both participate in processing facial identity and expression. On the one hand, FFA exhibited larger neural activity with increasing intensity in emotion stimuli, which is similar with the properties of pSTS (Calder and Young, 2016, Duchaine and Yovel, 2015). On the other hand, the pSTS was found to make contributions in identity recognition, especially to dynamic faces (Bernstein and Yovel, 2015). Furthermore, a vertical occipital fasciculus was traced between the face-selective region in the fusiform area and the dorsal visual pathway, which may provide a structural base for the interaction between FFA and pSTS (Yeatman et al., 2014, Grill-Spector et al., 2017). Thus, our finding about the co-lateralization of FFA and pSTS is supported by both functional and structural evidence.

Third, a strong and consistent relationship was observed between the FFA and OFA in terms of the estimated functional asymmetry. This is as expected because a solid consensus has been reached in different face processing models that the OFA provides input information to the FFA for invariant information processing after an early visual detection (Haxby et al., 2000, Bernstein and Yovel, 2015, Duchaine and Yovel, 2015). Besides, a robust structural connection exists between these two face-selective regions providing supporting for the between-ROI functional relationship (Gschwind et al., 2012; Liu et al., 2020, Liu et al., 2022). In terms of co-lateralization, a neuroimaging study found that the asymmetric property of the OFA mainly affected the right hemispheric dominance of the face system, including the lateralization of FFA at the higher functional level (Frässle et al., 2016). Generally, the well-validated connection between the FFA and OFA in multiple dimensions can well explain our finding. Importantly, our study extended previous findings from adults to children and validated the input asymmetry hypothesis in the face processing system.

In both correlation and path analyses, adults displayed weaker within-system co-lateralization than children. This is probably due to an increased functional specialization and differentiation through individual development. From a general perspective, the topological pattern of global functional sub-networks has the largest differentiation in young adulthood across lifespan when individual cognitive abilities are at the peak (Chan et al., 2014, Chan et al., 2017). Specific to the face system, regional specialization for decoding different dimensions of facial information also increases from children to adults (Kadosh and Johnson, 2007, Cohen Kadosh et al., 2011), accompanied by decreased areal association.

Similar with the face processing system, functional co-lateralization was observed in the VWFA, pSTG and Broca’s area during word and sentence reading. This is probably influenced by the overarching left hemispheric dominance in language processing, as demonstrated in previous studies (Cai et al., 2008, Cai et al., 2010, Van der Haegen et al., 2012). Given that the primary emphasis of our investigation is the intra-system relationship within the face processing system, a comprehensive discussion on co-lateralization within the reading system is beyond the scope of our study and will not be addressed herein.

### 4.4 Inter-system relationships for functional asymmetry in face processing and reading

For the inter-system relationship in functional lateralization of face processing and reading, both LI and PSC analyses demonstrated significant interactions across systems in adults but not children. To elaborate, analyses based on the LI revealed that the left lateralized Broca’s area significantly predicted the right lateralized FFA, while lateralization of the VWFA could positively predict that of the pSTS. For the PSC analysis, larger VWFA response in the left hemisphere during reading correlated with increased neural activity in the right-side FFA and pSTS during face processing. Relationships failing to pass multiple comparison correction were not involved in the discussion in case of overinterpretation.

While limited research has examined the maturational change in the inter-system correlation between face processing and reading across the developmental spectrum from children to adults, our identified negative relationship between the LI in the FFA and Broca’s area converges with the findings of Gerrits et al. (2020). Their study revealed that language dominance in the Broca’s area (Brodmann areas 44 and 45) was significantly correlated with the face recognition dominance in the occipital lobe and fusiform gyrus (Brodmann areas 19 and 37) with a medium effect size. Moreover, prior research found increasing reading performance to be associated with larger face responses in the right fusiform area (Dehaene et al., 2010, Pegado et al., 2014, Dehaene-Lambertz et al., 2018). Given that enhanced reading proficiency frequently coincided with an increased left-brain response in language processing regions (Dehaene-Lambertz et al., 2018, Feng et al., 2022), it is reasonable to posit that neural activation in left reading-related regions is interconnected with that in the right face-related regions, thereby corroborating our PSC findings. The collective evidence implies that the right-lateralized face processing could be influenced by the prevailing left hemisphere dominance in language processing, aligning with the causal complementarity hypothesis.

We found no significant interaction between face and reading systems in the child group. In terms of this, previous developmental studies generally exhibited mixed results. Several fMRI studies indicated that during the first few years of reading acquisition, the generation of VWFA among children did not influence the size or location of category-selective regions for faces in the ventral temporal lobe (Dehaene-Lambertz et al., 2018, Nordt et al., 2021, Feng et al., 2022). Additionally, an early behavioral study spanning multiple age groups, including children and teens, failed to identify a connection between the lateralization of visual processing for faces and words (Dundas et al., 2013). These results are consistent with our findings in children, suggesting the absence of interaction between face processing and reading systems in terms of functional asymmetry. Moreover, given the logographic and holistic processing features of Chinese characters, it is also plausible that children perceive these words more as pictures compared to adults. This consequently results in weaker inter- and intra-system correlations in the younger cohort. Notably, reading capacity of children were found to correlate with face responses in the right fusiform gyrus in a few studies (Dundas et al., 2013, Dehaene-Lambertz et al., 2018, Lochy et al., 2019), indicating a potential indirect effect from reading to face perception. Based on both adult and child studies discussed above, we propose that the influence from reading acquisition to face processing exists, but takes longer developmental time from children to adults becoming more evident.

One unexpected outcome was the positive relationship in the LI between the pSTS for face processing and the VWFA engaged in visual reading, not negative. This observation may be explained by writing skills which usually gets trained together with reading (Graham, 2020). On one hand, the visual perception of handwriting procedure not only affects letter identification, but also shapes the middle temporal (MT) visual area during long-term training (Schubert et al., 2018, Vinci-Booher and James, 2020). On the other hand, the MT region provides input for the pSTS in the face processing model of O'Toole et al. (2002). Therefore, a co-lateralization of the VWFA and the pSTS might arise from a shared influence from visual training in handwriting skills. Notably, a prior ERP study documented a negative relationship between the right lateralization of face N170 and left lateralization of Chinese word N170 at the bilateral temporal lobe (Li et al., 2013), providing support for the current findings. However, as limited studies have explored the impact of reading and writing on the dorsal pathway of face processing, this hypothesis remains to be tested in future research.

### 4.5 Connections to classical lateralization theories

Under the framework of the classical theories on brain functional asymmetry, our findings support the input asymmetry theory and the causal complementary theory, while partially contradicting the statistical complementary theory. Firstly, we confirmed distinct hemispheric dominance in face and text processing and observed functional co-lateralization of cortical regions across hierarchical neural processing stages within each system. This aligns with the input asymmetry theory (Sergent, 1983, Andresen and Marsolek, 2005), which suggests that the perception of visual stimuli with specific characteristics induces functional asymmetry at lower processing levels. This functional asymmetry may subsequently influence cortical functional lateralization at higher levels, evidenced by a positive correlation between the asymmetries of subsystems or intra-system brain regions. Secondly, we identified an interaction between the lateralization in the reading and face processing systems. This finding supports the causal complementarity theory (Bryden et al., 1983, Bryden, 1990), which proposes that the development of functional asymmetry in one system may lead to opposite asymmetry in its complementary system. Notably, a strict causal relationship should be further examined using more carefully designed and longitudinal experiments. Nonetheless, our current findings provide a solid foundation and valuable insights for future research. Thirdly, our results partially support the statistical complementarity theory (Bryden et al., 1983, Bryden, 1990) as inter-system independence was only observed among children. This finding suggests that a dynamic perspective is necessary when investigating properties of brain functional asymmetry.

Comparatively, the statistical complementarity theory emphasizes innate factors determining functional asymmetry, while the input asymmetry and causal complementarity theories suggest positive and negative influences from neighboring and homotopic regions or systems. Therefore, comparing the advantages and disadvantages of different lateralization theories may be less meaningful, since multiple mechanisms may coexist and vary across different developmental stages and different cognitive domains (Cai et al., 2013, Packheiser et al., 2020).

### 4.6 Limitations and future directions

Several considerations regarding limitations in the current study are essential. First, we observed disparities in the interaction of the reading system and the face processing system between adults and children. Given that reading and face processing capacities exhibit improvement throughout individual development, future research necessitates longitudinal studies spanning from preschool age to adulthood to comprehensively elucidate the mechanisms involved in functional specialization. Second, using dynamic face stimuli could enhance the exploration of the dorsal pathway. While prior studies confirm pSTS activation in response to static neutral faces, inclusion of emotional or short clips of faces may better align with its role in processing dynamic facial information and elicit larger neural responses. Third, despite the generally consistent results obtained through correlation and path analyses based on the LI and PSC computation, the current sample size remains limited. Subsequent endeavors may consider increasing participant numbers or validating analyses through available open-access databases. Fourth, the definition of target ROIs relied on an anatomical atlas and meta-analysis in a unified way. Future investigations can explore whether adopting individualized ROIs could impact the current results. Finally, as discussed above, investigating whether the acquisition of writing skills influences the dorsal pathway of face processing in dynamic information processing would be an intriguing topic. Such an investigation would provide valuable insights about how postnatal literacy skills may shape brain and behavior during individual development.

## 5 Conclusion

In sum, the present study investigated the developmental features of lateralization in the core face processing system and reading system, with a focus on potential within and between system contributions to the right hemispheric dominance of face perception. Our findings demonstrated that both young adults and school-aged children exhibited functional lateralization in visual processing of faces and texts. Moreover, brain regions at higher functional level exhibits stronger lateralization among all participants in the face system and among only adults in the reading system. In both age groups, intra-system relationships were identified for the lateralization of the FFA and pSTS. While the interaction between the face processing and reading clusters were exclusive to adults. Overall, the current investigation consolidates the neural competition theory from a developmental perspective and provides neuroimaging evidence from Chinese cohorts for the first time.

## CRediT authorship contribution statement

**Xinyang Liu:** Writing – review & editing, Writing – original draft, Visualization, Software, Methodology, Funding acquisition, Formal analysis, Conceptualization. **Danni He**: Writing – review & editing, Methodology, Data curation. **Miaomiao Zhu:** Methodology. **Yinghui Li:** Data curation. **Longnian Lin:** Writing – review & editing, Funding acquisition. **Qing Cai:** Writing – review & editing, Methodology, Funding acquisition, Formal analysis, Data curation, Conceptualization.

## Declaration of Competing Interest

The authors declare that they have no known competing financial interests or personal relationships that could have appeared to influence the work reported in this paper.

## Data Availability

Data will be made available on request.
