# Supplementary material for: Hemispheric dominance in reading system alters contribution to face processing lateralization across development
Source: Dev Cogn Neurosci. 2024 Jul 22;69:101418. doi: 10.1016/j.dcn.2024.101418 (PMC11331717; doi:10.1016/j.dcn.2024.101418)
Supplement: Supplementary file 1 — Supplementary material [file mmc1.docx]

**Supplementary Information**


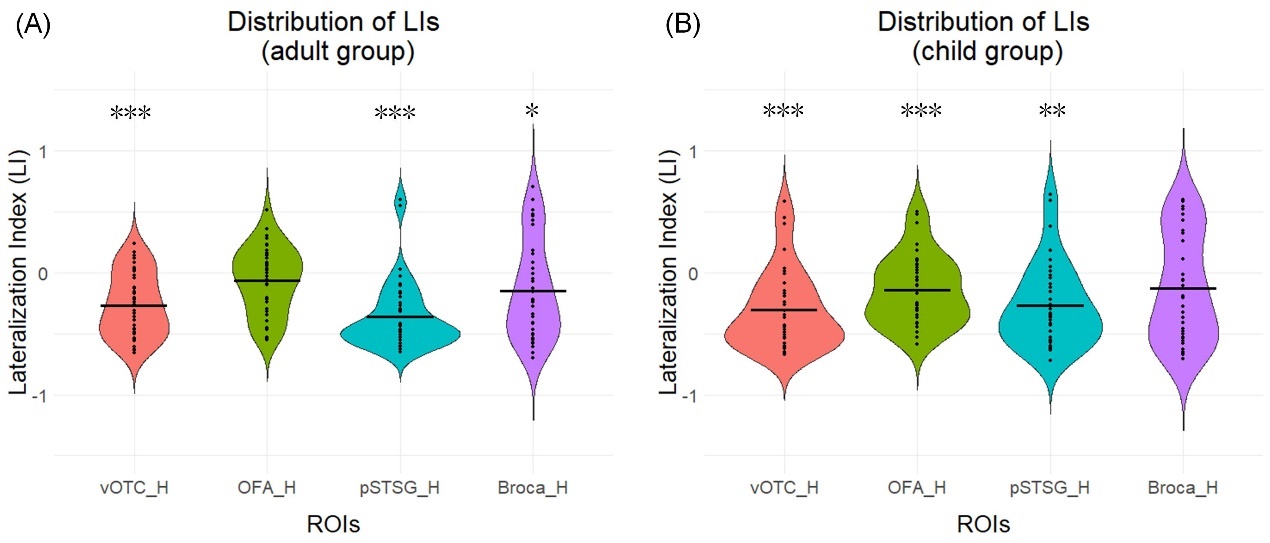


**Figure S1** Distribution of weighted mean LI values in the adult and child groups. (A) LI distribution of responses to houses in four target ROIs among adults. (B) LI distribution of responses to houses in four target ROIs among children. vOTC_H, OFA_H, pSTSG_H and Broca_H represent neural responses to *houses* stimuli in four target ROIs. **p*< 0.05, ** *p* < 0.01, *** *p* < 0.001, FDR corrected.

**Table S1**

Spearman correlation matrix for the adult group

|  | OFA_L | OFA_R | FFA_L | FFA_R | pSTS_L | pSTS_R | VWFA_L | VWFA_R | pSTG_L | pSTG_R | Broca_L | Broca_R |
| --- | --- | --- | --- | --- | --- | --- | --- | --- | --- | --- | --- | --- |
| OFA_L | *r* = 1 |  |  |  |  |  |  |  |  |  |  |  |
| OFA_R | ***r* = .704**  ***p* < .001** | *r* = 1 |  |  |  |  |  |  |  |  |  |  |
| FFA_L | ***r* = .669**  ***p* < .001** | ***r* = .478**  ***p* = .002** | *r* = 1 |  |  |  |  |  |  |  |  |  |
| FFA_R | *r*= .364*  *p* = .021 | ***r* = .415**  ***p* = .008** | ***r* = .557**  ***p* < .001** | *r* = 1 |  |  |  |  |  |  |  |  |
| pSTS_L | *r* = .101  *p* = .536 | *r* = .041  *p* = .802 | ***r* = .429**  ***p* = .006** | *r* = .160  *p* = .324 | *r* = 1 |  |  |  |  |  |  |  |
| pSTS_R | *r* = .136  *p* = .404 | *r* = .036  *p* = .824 | *r*= .329*  *p* = .038 | ***r* = .465**  ***p* = .003** | ***r* = .711**  ***p* < .001** | *r* = 1 |  |  |  |  |  |  |
| VWFA_L | *r* = .070  *p* = .667 | *r* = .001  *p* = .995 | *r* = .218  *p* = .176 | ***r* = .502**  ***p* = .001** | *r* = .267  *p* = .095 | ***r* =.411**  ***p* = .008** | *r* = 1 |  |  |  |  |  |
| VWFA_R | *r* =-.075  *p* = .646 | *r* = -.131  *p* = .420 | *r* = -.056  *p* = .733 | *r* = .274  *p* = .087 | *r* = .112  *p* = .490 | *r* =.348*  *p* = .028 | *r* = .640  *p* < .001 | *r* = 1 |  |  |  |  |
| pSTG_L | *r* = .075  *p* = .644 | *r* = .183  *p* = .259 | *r* = .225  *p* = .163 | *r* = .268  *p* = .095 | *r* = .149  *p* = .359 | *r* =.008  *p* = .961 | *r* = .242  *p* = .132 | *r* = .166  *p* = .306 | *r* = 1 |  |  |  |
| pSTG_R | *r* =-.023  *p* = .889 | *r* = .085  *p* = .603 | *r* = .103  *p* = .526 | *r* =-.030  *p* = .854 | *r* = .120  *p* = .461 | *r* =.013  *p* = .939 | *r* = -.061  *p* = .707 | *r* = .218  *p* = .177 | ***r* = .701**  ***p* < .001** | *r* = 1 |  |  |
| Broca_L | *r* = .036  *p* = .823 | *r* = .211  *p* = .191 | *r* = .120  *p* = .461 | *r*= .321*  *p* = .043 | *r* = .023  *p* = .889 | *r* =.104  *p* = .523 | *r* = .137  *p* = .400 | *r* = .037  *p* = .820 | ***r* = .591**  ***p* < .001** | *r* = .378*  *p* = .016 | *r* = 1 |  |
| Broca_R | *r* = .185  *p* = .254 | *r* = .247  *p* = .125 | *r*= .331*  *p* = .037 | *r* = .090  *p* = .580 | *r* = .124  *p* = .447 | *r* = -.032  *p* = .843 | *r* = -.129  *p* = .427 | *r* = -.013  *p* = .939 | ***r* = .556**  ***p* < .001** | ***r* = .640**  ***p* < .001** | ***r* = .698**  ***p* < .001** | *r* = 1 |

*Note.* Correlation coefficients and p values for pairwise PSC of bilateral face- and reading-related ROIs among adults. The displayed *p* values were uncorrected. **p* < 0.05 uncorrected, but did not reach significance after FDR correction. The *r* and *p* values shown in bold indicate significant results after FDR correction (FDR *q* < 0.05).

**Table S2**

Spearman correlation matrix for the child group

|  | OFA_L | OFA_R | FFA_L | FFA_R | pSTS_L | pSTS_R | VWFA_L | VWFA_R | pSTG_L | pSTG_R | Broca_L | Broca_R |
| --- | --- | --- | --- | --- | --- | --- | --- | --- | --- | --- | --- | --- |
| OFA_L | *r* = 1 |  |  |  |  |  |  |  |  |  |  |  |
| OFA_R | ***r* = .649**  ***p* < .001** | *r* = 1 |  |  |  |  |  |  |  |  |  |  |
| FFA_L | ***r* = .685**  ***p* < .001** | *r* = .387  *p* = .020 | *r* = 1 |  |  |  |  |  |  |  |  |  |
| FFA_R | ***r* = .500**  ***p* = .002** | ***r* = .693**  ***p* < .001** | ***r* = .691**  ***p* < .001** | *r* = 1 |  |  |  |  |  |  |  |  |
| pSTS_L | ***r* = .470**  ***p* = .004** | *r* = .297  *p* = .079 | ***r* = .597**  ***p* < .001** | *r* = .257  *p* = .131 | *r* = 1 |  |  |  |  |  |  |  |
| pSTS_R | *r* = .287  *p* = .089 | *r*= .359*  *p* = .031 | *r*= .395*  *p* = .017 | ***r* = .415**  ***p* = .012** | ***r* = .640**  ***p* <.001** | *r* = 1 |  |  |  |  |  |  |
| VWFA_L | *r* = -.039  *p* = .822 | *r* = .006  *p* = .970 | *r* = .077  *p* = .655 | *r* = .304  *p* = .072 | *r*= -.040  *p* = .817 | *r* = -.093  *p* = .591 | *r* = 1 |  |  |  |  |  |
| VWFA_R | *r* = .329  *p* = .050 | *r* = .246  *p* = .149 | *r* = .199  *p* = .245 | *r* = .317  *p* = .060 | *r*= -.147  *p* = .391 | *r* = -.356*  *p* = .033 | ***r* = .615**  ***p* < .001** | *r* = 1 |  |  |  |  |
| pSTG_L | *r* = .034  *p* = .843 | *r* = .088  *p* = .611 | *r* = -.018  *p* = .915 | *r* = .037  *p* = .830 | *r* = .216  *p* = .205 | *r* = .045  *p* = .794 | *r* = .200  *p* = .242 | *r* = .190  *p* = .267 | *r* = 1 |  |  |  |
| pSTG_R | *r* = .131  *p* = .448 | *r* = .221  *p* = .194 | *r* = .051  *p* = .766 | *r* = .145  *p* = .398 | *r* = .179  *p* = .296 | *r* = .123  *p* = .475 | *r* = .068  *p* = .695 | *r* = .222  *p* = .194 | ***r* = .778**  ***p* < .001** | *r* = 1 |  |  |
| Broca_L | *r* = -.092  *p* = .593 | *r* = -.108  *p* = .531 | *r* = -.110  *p* = .524 | *r* = -.080  *p* = .643 | *r* = .175  *p* = .308 | *r* = -.013  *p* = .939 | *r* = .168  *p* = .326 | *r* = .113  *p* = .511 | ***r* = .843**  ***p* < .001** | ***r* = .682**  ***p* < .001** | *r* = 1 |  |
| Broca_R | *r* = .167  *p* = .332 | *r* = .183  *p* = .287 | *r* = -.042  *p* = .809 | *r* = .053  *p* = .758 | *r* = .078  *p* = .651 | *r* = -.017  *p* = .923 | *r* = .028  *p* = .871 | *r* = .330*  *p* = .049 | ***r* = .625**  ***p* < .001** | ***r* = .802**  ***p* < .001** | ***r* = .671**  ***p* < .001** | *r* = 1 |

*Note.* Correlation coefficients and p values for pairwise PSC of bilateral face- and reading-related ROIs among children. The displayed *p* values were uncorrected. **p* < 0.05 uncorrected, but did not reach significance after FDR correction. The *r* and *p* values shown in bold indicate significant results after FDR correction (FDR *q* < 0.05).


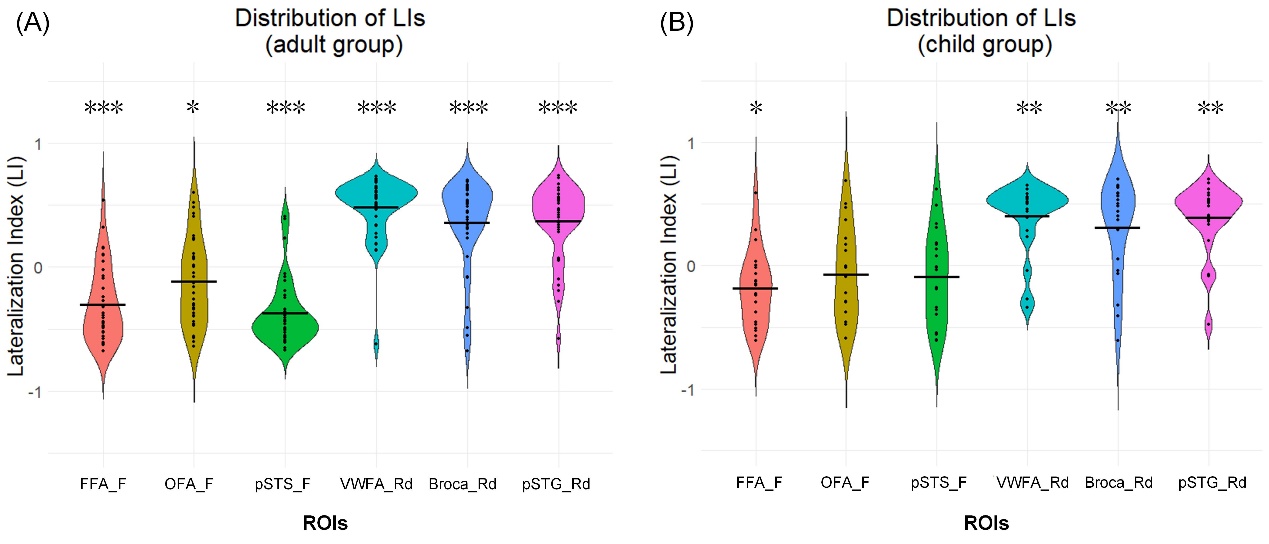


**Figure S2** Distribution of weighted mean LI values in the adult and child groups with house stimuli as the contrast baseline. (A) LI distribution of all target ROIs in the adult group. (B) LI distribution of all target ROIs in the child group. FFA_F, OFA_F, pSTS_F represent three core regions in the face processing system. VWFA_Rd, Broca_Rd and pSTG_Rd indicate three crucial ROIs in the reading system. **p*< 0.05, ** *p* < 0.01, *** *p* < 0.001, FDR corrected.
